# Enhancing Dialysis Access: A Journey in Advocacy and Patient Care

**DOI:** 10.34067/KID.0000000000000411

**Published:** 2024-03-22

**Authors:** Prakrati Acharya

**Affiliations:** Nephrology, Texas Tech University Health Sciences Center, El Paso, Texas

**Keywords:** chronic hemodialysis, hemodialysis access, patient-centered care, quality of life, social health justice

“Doctor, please do something!” cried Ms. AS, her desperate plea echoing through the emergency department (ER) as she grappled with worsening shortness of breath. “Is there any way I can have permanent dialysis access and avoid the agony of getting a new catheter each time?” I sympathized with her, feeling an equal sense of helplessness!!

In the hospitals of our border city, the protocol for patients declared to have ESKD requires consulting social workers to determine insurance or Medicare eligibility. If the patients do not meet the criteria, they are denied access to a tunneled catheter and arteriovenous fistula (AVF). Instead, they get a temporary catheter during each ER visit, further adding to their torment.

I could not turn a blind eye to the heart-wrenching scenes. Patients on the brink of death, got a temporary hemodialysis catheter, received 2–3 dialysis sessions, and got the catheter removed before discharge—this became a routine, tugging at my heartstrings 3–5 times a week. Some patients faced more difficulties because of the veins damaged by numerous prior catheters.

Distraught after the call with Ms. AS, I contacted my chief. I expressed my growing inability to witness these scenes and inquired about potential solutions. Our conversation delved into his past efforts to bring about change, recalling a time when he, alongside a vascular surgeon, faced repercussions for challenging the *status quo* two decades ago. “Talk to the new administration,” he advised, acknowledging the possibility of a different outcome.

The next day, I met with the administrators of the hospital. A glimmer of hope emerged as a new vascular surgeon was willing to lend support. Despite knowing that opting for a fistula would mean a few more months of temporary catheter reliance, I weighed the benefits—reduced infections and shorter hospitalizations—and decided it was a more sustainable and cost-effective choice than a tunneled dialysis catheter. After meetings, the hospital administration approved placing five AVF initially.

The goal was to increase this number on the basis of the experience and financial implications. Each time a patient seemed a candidate for permanent access, we notified case management, and more often than not, the requests were approved. By the end of the year, about half of the patients got approval for AVF placement before enrolling in a private health insurance program available through a philanthropic organization for regular dialysis. It was a significant triumph for our patients, reinforcing the importance of persistent patient advocacy. Me and my patients are thankful to the hospital administration. Even if doors were shut in the past, it is always worth knocking again!

I drew inspiration from the work of Dr. Lilian Cervantes, reaching out to her for guidance. Her words, “One person can make a difference (you!),” became my mantra, propelling me forward. In the current system, patients younger than 65 years who are not eligible for Medicare or are uninsured, underwent emergent dialysis through the ER from January to November each year. During November through December, they got enrolled for regular dialysis under insurance programs supported by philanthropic organizations, for which we are immensely grateful.

Our hospital maintained a year-round list, and nephrologists enrolled CKD stage 5 patients in this program. The aim was to prevent emergent dialysis through the ER, but challenges persisted with crash start patients and those lost to regular follow-up. The so-called compassionate dialysis, provided under the Emergency Medical Treatment And Labor Act, fell short of truly embodying compassion. Patients on emergent dialysis experience significantly higher mortality rates, with undocumented immigrants facing a staggering 14 times the mortality rate compared with those on scheduled dialysis.^1^ The toll on health care providers was evident, acknowledging the substandard care they were compelled to provide. Moreover, it was economically inefficient for the system.

Despite evidence, several states lag in acknowledging the need for policy changes. Colorado's shift, including ESKD as a qualifying condition for emergency Medicaid, showcased potential savings of 19 million dollars.^2^ Slowly, 20 states have adopted provisions to provide emergency Medicaid to these patients.^3^

Undeterred and inspired by successful programs in Texas, I revisited the hospital administration, advocating for the establishment of a peritoneal dialysis (PD) program.

Extensive research, collaboration with hospital departments, and engaging potential participants laid the groundwork for the proposed program. Our hospital did not have its own outpatient dialysis unit, thus requiring a new setup. I reached out to our billing/financial division to understand the current expenditure and reimbursement scenario. I also reached out to the pharmacy to understand the cost of drugs and dialysis company to get a price quote for PD supplies. We needed a nurse who was qualified to do PD. After contacting many nurses, I was able to identify a nurse who was willing to help us part time. I also got feedback from potential patients. Significant time and energy was invested to make a plan, although I was unsure all the time whether all my commitment to set up a PD program would be a reality, but I kept going to do my part.

The final plan centered around continuous ambulatory PD at home. Patients would receive training at home, and all necessary supplies would be delivered directly to their residences. The patient would be seen in clinic by nephrologists monthly with as-needed nursing visits.

The suggestion was that the hospital would place a PD catheter and provide a intravenous pole, ultra-clamp, 2 L dialysis bags, Y set, minicaps, transfer sets, and nursing care. Refurbished cyclers would be an option for high transporters only. Patients would be responsible for soap, hand sanitizer, BP cuff, gauze box, tape, towels, gloves, spring scale, weight scale, heating pad, and disinfectant.

After cost of PD catheter placement, the calculated costs for supplies and nursing support for the hospital for three cycles/day for 5 days a week came out to be $ 32,500/yr per patient or $2700/mo per patient. While the same for three cycles for 7 days per week would have been $45,260/yr per patient or $3800 per month per patient. Laboratory tests and medications including calcimimetics, antibiotics, heparin, binders, and iron would be additional.

Even if this did not cover every need—it was much more supported care than what our patients currently get through recurrent hospitalizations.

I presented this to our hospital administration again. I felt heard and appreciated the hospital's effort. They made a team of hospital administrators to look further into this. We had a series of meetings. The presentation to the hospital administration showcased the potential benefits, emphasizing improved patient care and reduced hospitalization rates and costs.

However, concerns about investments (especially with no existing hospital owned dialysis unit), uncertain home conditions, and potential patient influx led to hesitation to set up a PD program.

Hospitals currently do not have incentives from state or central programs to support an outpatient dialysis program for patients meeting criteria for emergency dialysis. Hospitals get reimbursement during these hospitalizations through emergency in hospital Medicaid. It would have been profitable for the hospital to invest money if the beds occupied by patients undergoing emergent dialysis were available to insured patients, but it was hard to predict whether the insurance status of an incoming patient. Hospitals not owning dialysis units may need to invest significant resources to set up an outpatient dialysis program. PD is cheaper compared with hemodialysis, but there may be patients, especially in this unfunded, undocumented cohort, whose home conditions are not conducive. For community hospitals in medium to smaller cities that have a limited number of unfunded patients, it might not be a financially viable option to open a dialysis unit where these patients could be served.

While acknowledging the support from philanthropic programs, uncertainties about their year-round availability persist. There is not a program available for patients older than 65 years. The need for continued advocacy, engaging with senators and the government, and leveraging organizations, such as American Society of Nephrology/National Kidney Foundation, became evident to drive systemic change.

Despite the challenges, progress was made with an increased approval of permanent access for patients. However, the struggle for regular dialysis in the border town and states persists. While unsuccessful in setting up regular dialysis, I find solace that we could make some positive impact for my patients.

In conclusion, while challenges persist, my commitment to patient advocacy remains unwavering. The hope is that, with collective efforts and continued advocacy, systemic changes will pave the way for a future where every patient receives the standard of care they deserve.

## Supplementary Material
