# Supplementary material for: Enhancing Dialysis Access: A Journey in Advocacy and Patient Care
Source: Kidney360. 2024 Mar 22;5(6):907–8. doi: 10.34067/KID.0000000000000411 (PMC11219115; doi:10.34067/KID.0000000000000411)
Supplement: Supplementary file 1 [file kidney360-5-907-s001.pdf]

ASN Journal Disclosure Form

As per ASN journal policy, I have disclosed any financial relationship or commitment held by myself and/or my spouse/partner in the past 36 months as included below. I have listed my Current Employer below to indicate there is a relationship requiring disclosure. If no relationship exists, my Current Employer is not listed.

P. Acharya reports the following:
Other Interests or Relationships: Member of ASN Health Care Justice Committee

I understand that the information above will be published within the journal article, if accepted, and that failure to comply and/or to accurately and completely report the potential financial conflicts of interest could lead to the following: 1) Prior to publication, article rejection, or 2) Post-publication, sanctions ranging from, but not limited to, issuing a correction, reporting the inaccurate information to the authors' institution, banning authors from submitting work to ASN journals for varying lengths of time, and/or retraction of the published work.

Name: Prakrati C. Acharya
Manuscript ID: K360-2024-000143
Manuscript Title: "Enhancing Dialysis Access: A Journey in Advocacy and Patient Care"
Date of Completion: February 26, 2024
Disclosure Updated Date: February 26, 2024
